# Probing the multi-disordered nanoscale alloy at the interface of lateral heterostructure of MoS_2_–WS_2_

**DOI:** 10.1515/nanoph-2023-0826

**Published:** 2024-01-19

**Authors:** Dong Hyeon Kim, Chanwoo Lee, Sung Hyuk Kim, Byeong Geun Jeong, Seok Joon Yun, Hyeong Chan Suh, Dongki Lee, Ki Kang Kim, Mun Seok Jeong

**Affiliations:** Department of Physics, Hanyang University, Seoul 04763, Korea; Department of Energy Science, Sungkyunkwan University, Suwon 16419, Korea; Department of Semiconductor, University of Ulsan, Ulsan 44610, Republic of Korea; Department of Nanotechnology and Advanced Materials Engineering, Sejong University, Seoul 05006, Korea

**Keywords:** tip-enhanced Raman spectroscopy (TERS), molybdenum disulfide (MoS_2_), tungsten disulfide (WS_2_), multi-disorder, nanoscale alloy

## Abstract

Transition metal dichalcogenide (TMDs) heterostructure, particularly the lateral heterostructure of two different TMDs, is gaining attention as ultrathin photonic devices based on the charge transfer (CT) excitons generated at the junction. However, the characteristics of the interface of the lateral heterostructure, determining the electronic band structure and alignment at the heterojunction region, have rarely been studied due to the limited spatial resolution of nondestructive analysis systems. In this study, we investigated the confined phonons resulting from the phonon-disorder scattering process involving multiple disorders at the lateral heterostructure interface of MoS_2_–WS_2_ to prove the consequences of disorder-mediated deformation in the band structure. Moreover, we directly observed variations in the metal composition of the multi-disordered nanoscale alloy Mo_1−*x*_W_*x*_S_2_, consisting of atomic vacancies, crystal edges, and distinct nanocrystallites. Our findings through tip-enhanced Raman spectroscopy (TERS) imply that a tens of nanometer area of continuous TMDs alloy forms the multi-disordered interface of the lateral heterostructure. The results of this study could present the way for the evaluation of the TMDs lateral heterostructure for excitonic applications.

## 1 Introduction

The widespread investigation of two-dimensional (2D) layered transition metal dichalcogenides (TMDs) has garnered significant attention due to their exceptional physical properties [1]–[5]. The distinctive features arising from the direct band gap of monolayer TMDs within the visible to near-infrared range offer opportunities for modulating band offset and band gap [6]. Particularly noteworthy are the unique behaviors of quasi-particles resulting from the quantum confinement effect in the vertical direction, which positions TMDs as pivotal in advancing next-generation quantum engineering applications such as optoelectronics, spintronics, and valleytronics [7]–[12].

Semiconductor heterojunctions, consisting of two different materials, play a crucial role in actively controlling charge carrier behaviors. Through van der Waals stacking of two dissimilar TMDs [13], researchers have fabricated vertical heterostructure semiconductors with type-II band alignment (staggered), enabling the modulation of carrier flow at the interface through an internal field [14], [15]. Vertical heterostructure TMDs can generate charge transfer (CT) excitons [16], [17] in the out-of-plane direction, which can be manipulated with bias voltage. Furthermore, lateral heterostructure TMDs have been extensively studied, leveraging covalently bonded edge contacts in the in-plane structure to easily control the electronic band structure and alignment with no dielectric gap [6], [17]. However, the interfaces of lateral heterostructure TMDs exhibit multiple disorders, including atomic vacancies, substitutions, nanocrystallites, nanoscale alloys, etc., which disrupt the application of heterostructure of a semiconductor due to the inducing of distortion at the electronic band structure [6], [18], [19]. To investigate the interface nature of lateral heterostructure TMDs, several studies have employed confocal microscopy-based photoluminescence (PL) and Raman spectroscopies. Despite the spatially resolved spectroscopic information, the optical diffraction limit constrains the exploration of heterojunction interface characteristics at the nanoscale. While recent studies have reported on exciton and phonon behavior at the nanoscale using near-field scanning optical microscopy (NSOM) and tip-enhanced Raman spectroscopy (TERS) [20]–[24], there is a lack of research on alloy composition changes with various disorders at the nanoscale.

In this study, we utilized scanning tunneling microscopy (STM)-based TERS to investigate the interface nature of the lateral heterostructure of MoS_2_–WS_2_. TERS measurements at 10 nm intervals made it possible to directly observe alloy composition changes and disorder-related phonon properties in the nanoscale heterojunction region. The multispectral information obtained through TERS, reflecting multi-disordered continuous transition metal composition changes, provides valuable insights for understanding and applying the interfacial phenomena of the lateral heterostructure of MoS_2_–WS_2_ based on the clue to figure out the local electronic band structure for excitonic applications.

## 2 Methods

### 2.1 Synthesis of monolayer MoS_2_–WS_2_ lateral heterostructure

The monolayer MoS_2_–WS_2_ lateral heterostructure was synthesized by an atmospheric chemical vapor deposition (CVD) process. In order to synthesize the heterostructure, the precursor solution was prepared by mixing four different chemical solutions of *W* precursor, Mo precursor, promoter, and medium solution. First, the *W* precursor was fabricated by dissolving 0.1 g of ammonium metatungstate hydrate ((NH_4_)_6_H_2_W_12_O_40_·xH_2_O, Sigma–Aldrich) in 10 ml of DI water. Second, the Mo precursor was fabricated by dissolving 0.1 g of the ammonium heptamolybdate tetrahydrate ((NH_4_)_6_Mo_7_O_24_·4H_2_O, Sigma–Aldrich) in 10 ml of DI water. Third, the promoter was fabricated by dissolving 0.1 g of sodium hydroxide (NaOH, Sigma–Aldrich) in 30 ml of DI water. Lastly, the medium solution of iodixanol solution (Sigma–Aldrich) was used to mix the metal precursors with the promoter. The four different solutions were mixed in a ratio of 1 (W): 1 (Mo): 3 (NaOH): 0.5 (iodixanol), then was spin-coated on a SiO_2_/Si substrate with 3000 rpm for 1 min. The two-zone CVD furnace was used to control the temperature of the S and the substrate zone independently. The pure S and the precursor coated substrate were introduced into the upstream S zone and the downstream substrate zone, respectively. The temperature of the S zone is elevated to 220 °C at 50 °C min^−1^ while the substrate zone was ramped to 800 °C at 100 °C min^−1^ with flowing N_2_ (500 sccm) and H_2_ (5 sccm) gases. After 10 min of growth, both furnaces are opened and naturally cooled to room temperature.

### 2.2 TERS tip fabrication using electrochemical etching

The gold nano tip utilized for TERS measurements was produced through electrochemical etching [25], [26]. In this process, a gold wire (diameter of 250 μm, purity of 99.95 %, Nilaco) served as the anode and was connected to a wave function generator. The generator applied a square-wave voltage ranging from a minimum of −25 mV to a maximum of 3.5 V, with a frequency and duty cycle of 300 Hz and 20 %, respectively. Acting as the cathode in the etching process, a ring-shaped platinum wire (with a diameter of 200 μm, purity of 99.98 %, Nilaco) was immersed in an etchant comprising a 37 % HCl solution and 99.5 % anhydrous ethanol. Following the self-terminating etching process, the resulting TERS gold nano tip underwent rinsing with acetone, ethanol, DI water, and IPA solutions.

### 2.3 STM-based TERS measurements

The TERS system (NTEGRA Spectra, NT-MDT) is composed of both scanning tunneling microscopy (STM) and a confocal Raman scattering system. Preceding the TERS scanning process, STM imaging was employed to scrutinize the interface of the lateral heterostructure MoS_2_–WS_2_ monolayer. The STM images were acquired under specific scanning conditions, with a tunneling current of approximately 6 nA and a bias voltage of 0.1 V, within an ambient environment. The TERS scanning procedure utilized an excitation laser with a wavelength of 632.8 nm and an objective lens possessing a numerical aperture (NA) of 0.7 (Mitutoyo). Multispectral TERS spectra were acquired through a spectrometer featuring 1800 grooves/mm grating, blazed at 500 nm, and a CCD (Andor) cooled to a temperature of −80 °C. The same gold nano tip was used for all STM and TERS measurements.

## 3 Results and discussion

### 3.1 STM and TERS characterization of monolayer lateral heterostructure MoS_2_–WS_2_

**Figure 1:**
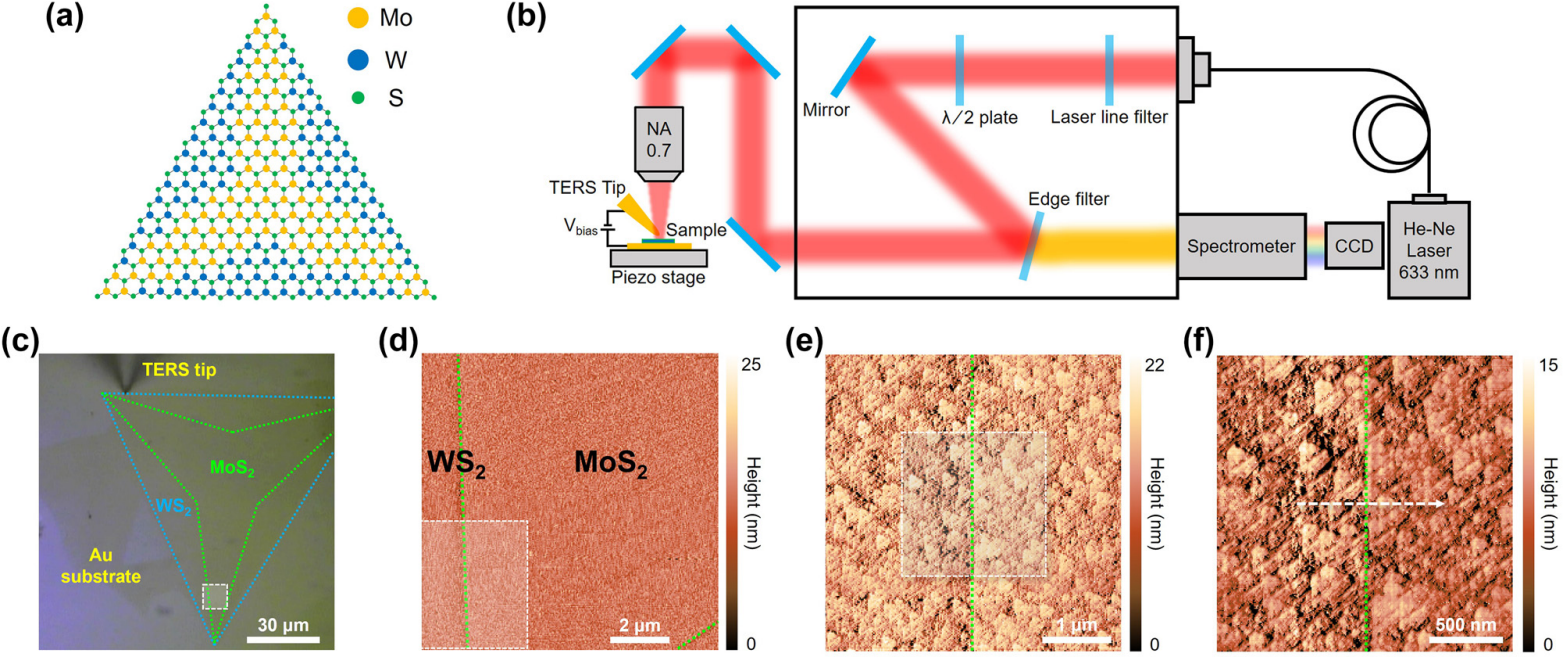
STM-TERS measurement for the lateral heterostructure TMD. (a) Schematic illustration of the lateral heterostructure of MoS_2_–WS_2_. (b) Schematic of STM-based TERS system. (c) Optical microscope image of a sample. (d–f) STM images for the interface of the lateral heterostructure MoS_2_–WS_2_. The white dashed squares in (c–e) indicate the STM imaging area of the fore figures, respectively. The white dashed arrow in (f) indicates the TERS scanning range and direction. The green dotted lines in (d–f) indicate the interface in the lateral heterostructure.

The monolayer lateral heterostructure of MoS_2_–WS_2_ by CVD process has been synthesized to investigate the interfacial nature of lateral heterostructure TMDs. As shown in Figure 1a, the Mo, W, and S atoms are covalently bonded, which can generate an intrinsic p-n heterojunction as an in-plane structure [8], [19]. The synthesis conditions and transition metal reactivity differences of each TMDs induced the laterally separated structure of MoS_2_–WS_2_. The wet-transferred synthesized lateral heterostructure semiconductors on a flat Au substrate were prepared to perform the STM measurements of the heterostructure interface. The angle between the nanotip and a normal of the prepared sample was precisely controlled to prevent tip drift issues during STM imaging and TERS scanning and to increase the degree of Raman signal enhancement. To control the position of the nanotip, the tunneling current was measured between the wired nano tip and Au substrate by applying a bias voltage [27], [28], [29]. Firstly, we measured the wide-area STM topography, which includes the heterostructure interface, and the scanning area is marked with a white dashed square in the optical microscope image (Figure 1c). In order to visualize the interface of the lateral heterostructure in detail, it was magnified that the area of the interfacial region identified with OM image to 2 μm × 2 μm by STM imaging. Also, we conducted TERS line trace measurement from WS_2_ to MoS_2_ region for 1um with 10 nm interval to investigate minutely the lattice vibration characteristics in the region of lateral heterostructure interface. The white dashed arrow in Figure 1f indicates the TERS line trace region and direction.

**Figure 2:**
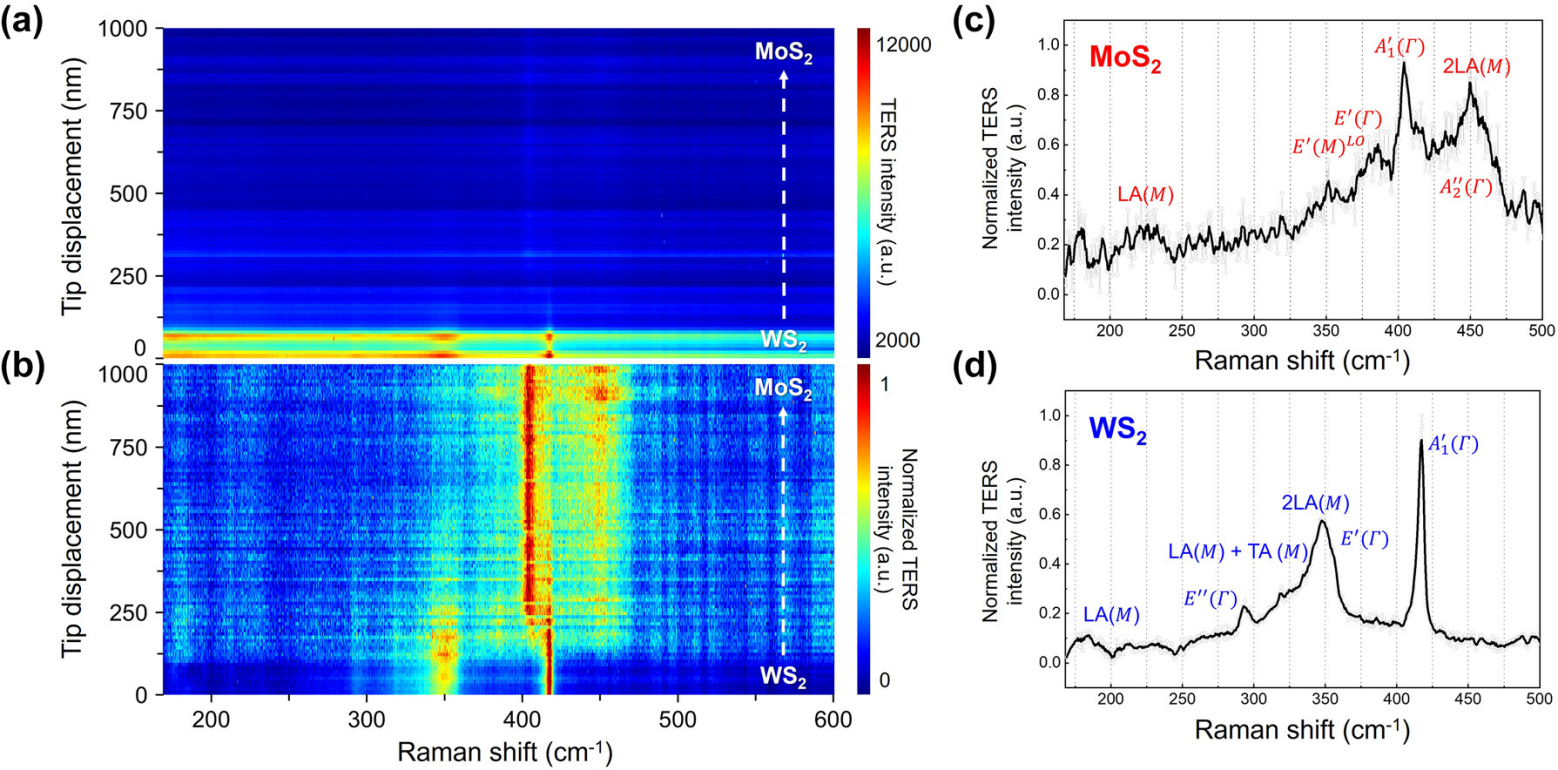
TERS scanning across the interface in the lateral heterojunction. (a) TERS multispectral line trace along the white dashed arrow in Figure 1f. (b) Normalized TERS spectra of (a) with background subtraction. Signature TERS spectra for monolayer (c) MoS_2_ and (d) WS_2_. The representative phonon modes of each material are marked.

Figure 2a shows the raw TERS spectra with PL background acquired by TERS line trace measurement. As the 632.8 nm (∼1.96 eV) laser excitation source well fitted the resonance conditions with the ∼1.94 eV of A excitonic absorption for WS_2_ (∼1.87 eV for MoS_2_), the strong resonance effect (semi-resonance effect) could enormously enhance the Raman scattering signal, and it leads the TERS intensity and SNR differences between WS_2_ and MoS_2_ regions [30], [31], [32], [33]. In order to figure out the variation of resonance Raman spectra at a glance, we normalized the PL (the tail of the WS_2_ PL) and plasmon (from the gap-mode localized surface plasmon resonance, LSPR) background signal subtracted from the TERS spectra (Figure 2b). Figure 2c and d are the representative TERS spectra of each TMDs material, and well-known phonon modes, including first-order modes, are marked. For convenience, we used red and blue colors to represent the MoS_2_ and WS_2_ phonon modes, respectively, based on the band gap. From the frequency difference between *E*′(Γ) and $A_{1}^{\prime }\left(\Gamma \right)$ of MoS_2_ and WS_2_, it is confirmed that both semiconductor materials are monolayer (Figure S1, Supplementary Materials). In addition, the first-order Raman scattering modes from the center of the Brillouin zone around 354, 385, 405, and 417 cm^−1^ in Figure 2b show drastic changes in both Raman intensity and phonon mode frequency near the interface of the lateral heterostructure.

### 3.2 Multi-disordered interface with confined phonon

**Figure 3:**
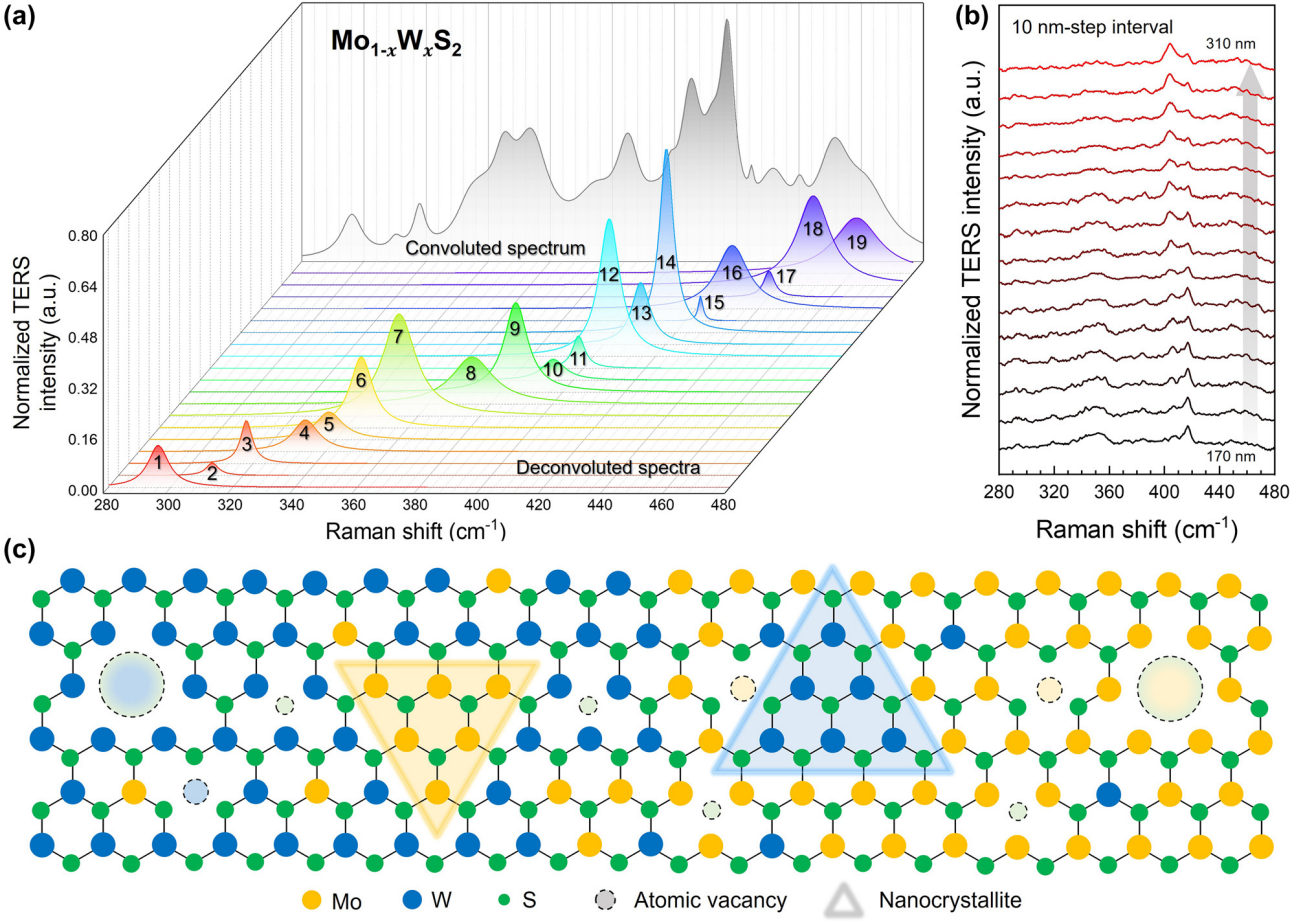
Diverse phonon modes at the interface of the lateral heterostructure with different origins. (a) Representative TERS spectrum of Mo_1−*x*_W_*x*_S_2_ at the interface of the lateral heterostructure. The gray spectrum indicates the convoluted spectrum of nineteen deconvoluted spectra. (b) Normalized TERS spectra extracted from Figure 2b. Tip displacements are marked with the gray arrow. (c) Schematic illustration of lateral heterostructure interface with multi-disorder. The dashed circles and highlighted triangles indicate the atomic vacancy and the nanocrystallites, respectively.

Although the TERS spectra in Figure 2b show significant fluctuations along the tip displacement, especially in the interface region of the lateral heterostructure, it is difficult to distinguish the change in phonon modes in detail because numerous phonon modes were observed due to the strong resonance effect of the tip-enhanced resonance Raman scattering (TERRS). The resonance Raman scattering process allowed the observation of the second-order Raman scattering signals, which it is unable to measure with non-resonant conditions. Thus, the strong LSPR owing to the nanocavity between the Au nanotip and the mirror image of the nanotip in the Au substrate greatly enhanced the weak Raman scattering signal (Figure S2, Supplementary Materials). In order to investigate the details of phonon modes, we assigned the deconvoluted spectra of the TERS spectrum from the interfacial region along the tip displacement from 170 nm to 310 nm, which shows drastic variation in the TERS spectra. Including the first- and second-order Raman scattering modes, nineteen phonon modes are convoluted in the range of 280 cm^−1^ to 480 cm^−1^. Figure 3a briefly presents the deconvolution result of the phonon modes of the Mo_1−*x*_W_*x*_S_2_ alloy with a representative TERS spectrum. The deconvoluted spectra were summarized in Table S1 (Supplementary Materials) with the corresponding phonon mode and frequency from the experimental results and previous reports. The numbers marked on each spectrum in Figure 3a are matched with the peak number in Table S1, and nineteen deconvoluted spectra contain information related to the multi-disorders, such as atomic vacancies, substitutions, line defects, alloys, and nanocrystallites [30], [34], [35], [36]. Also, not only the phonon modes that could be observed by far-field Raman spectroscopy but also forbidden phonon modes are accompanied in the deconvolution process. For the sake of convenience, the deconvoluted spectra in Figure 3a were categorized into four. As mentioned above, the phonon modes with red color and blue color indicate the vibrational modes of monolayer MoS_2_ and the monolayer WS_2_, respectively. First, the first-order Raman modes originating from the center of the Brillouin zone, Γ-point, were observed (peak 1, 7, 9, 12, 14, 19) [36], [37], [38]. These fundamental phonon modes are usually used to identify the existence of respective TMDs and the layer number of TMDs. Second, the strong resonance effect between the laser excitation source and the A excitonic absorption could lead to the observation of the combination of longitudinal acoustic (LA) and transverse acoustic (TA) phonon modes at the M point of the Brillouin zone (peak 4), and the overtone of the LA mode at the M point of the Brillouin zone (peak 6) [38], [39], [40]. We also observed the longitudinal optical (LO) phonon, the M point mediated by the disorders, which may originate from the double resonance process of MoS_2_ (peak 8) [35], [38]. Third, the commonly reported disorders and atomic vacancy-mediated phonon modes were also observed. The sulfur vacancies of WS_2_ led to symmetry breaking and allowed us to observe the *D* mode and *D*′ mode at the interface of the lateral heterostructure, which shows the same results with our previous research (peak 13, 16) [30], [34], [41]. Besides, for the MoS_2_, the vacancy of Mo, S, and MoS_6_ related phonon modes were calculated by density functional theory (DFT) before, there is only rare observation by the peak broadening and appearance of the shoulder of these phonon modes with conventional methods. However, we observed both separated to easily distinguishable vacancy-related modes and shoulders from the other phonon modes from the TERS spectra near the interface region (peak 11, 15, 17) [42]–[46]. Finally, we observed the evidence of the presence of the nanocrystallites of MoS_2_ and WS_2_ (peaks 2, 3, 5, 18) [35], [36]. Thus, the combination of LA and TA modes at the M point of MoS_2_ could be induced by the crystal-edge-related phonon (peak 10) [47]. These phonon modes change drastically along the tip displacement in the interfacial region (Figure 3b) because the observed Raman signals are the information of an ensemble of electric fields from the vicinity of tip-apex due to the high spatial resolution of TERS. Thus, both the phonon frequency and intensity could be easily influenced by the disorder density and nanocrystallite domain size. Furthermore, Figure 3c is a brief sketch of the concept of the multi-disordered interface of lateral heterostructure that could help catch the deconvoluted results at a glance.

For the most part, the origin of multi-disorder-related phonon modes can be explained by the phonon confinement effect. The phonon confinement effect is a kind of momentum conservation rule for the phonon-disorder scattering process, which could induce the relaxation of the Raman selection rule through the phonon weighting function. When the momentum conservation is required, *q* ≅ 0, where *q* is the momentum wave vector of the lattice vibration, the first-order Raman scattering can be introduced that can induce the zone center (Γ point)-related phonon from ideal crystalline materials without disorder. However, where the disorders are induced in the crystalline materials internally and/or externally, crystal structure would be broken and produced various nanostructures that contain multi-disorder such as atomic vacancy, nanocrystallites, and alloy. So, the disordered crystal with a finite phonon correlation length (*L*_*c*_) different from the pristine crystal (*L*_*c*_ ≅ ∞) could show the relaxation of the principal selection rule (*q* ≅ 0) for the Raman scattering process and introduce the lattice vibration away from the center of the Brillouin zone. In other words, the multi-disorders induced the selection-rule breaking to observe the forbidden phonons. The following phonon weighting function and the phonon confinement model by Richter–Wang–Ley (RWL model) can explain the relaxation process in detail [34], [35], [36],

(1) $$W\left(r,L_{c}\right)=\exp\left(-ar^{2}/L_{c}^{2}\right)$$

(2) $$I\left(\omega \right)\propto \int \frac{|C\left(q\right){|}^{2}}{{\left(\omega -\omega \left(q\right)\right)}^{2}+{\left(\Gamma_{0}/2\right)}^{2}}\text{d}q$$

(3) $$I\left(\omega \right)=\int \frac{\exp\left(-q^{2}L_{c}^{2}/2\alpha \right)}{{\left(\omega -\omega \left(q\right)\right)}^{2}+{\left(\Gamma_{0}/2\right)}^{2}}2\pi q\text{d}q$$

where *α* is an alterable confinement coefficient representing an attenuation of the lattice vibration amplitude, *I*(*ω*) is the intensity of the first-order Raman mode of the specific system, *C*(*q*) is the Fourier coefficient of the weighting function *W*(*r*, *L*_*c*_), $\omega \left(q\right)$ is the phonon dispersion curve in the infinite domain caused by disorders, and Γ_0_ is the width of the phonon peaks. The integral means the integration over the whole range of the Brillouin zone. For Eq. (3), not only *q* ≅ 0 phonons but also *q* ≠ 0 phonons can be involved in the Raman scattering process for the phonon confinement model, the phonon confined interface with atomic vacancies and nanostructures with nanocrystallites with some domains, *L*_*c*_. In addition, it is complicated to “see” the nature of these tiny nanostructures without the powerful TERRS.

**Figure 4:**
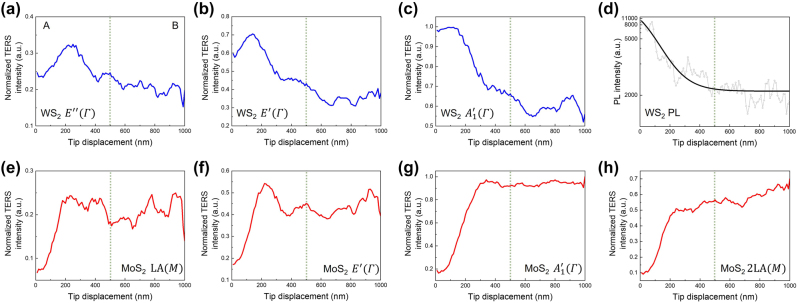
Tip-enhanced optical signal variation along the TERS line trace. Tip-enhanced optical signal line profiles along the white dashed arrow in Figure 1f correspond to (a–c) WS_2_ phonon modes (blue solid line), (d) photoluminescence background (black solid line) and (e–h) MoS_2_ phonon modes (red bold line). The domains A and B in (a) indicate the WS_2_ and MoS_2_ regions, respectively.

In Figure 4, we plotted the tendency of the prominent Raman scattering signals of each semiconductor along the tip displacement. The green dashed lines indicate the lateral heterostructure interface, as confirmed by the OM and STM images (Figure 1c–f, respectively). In accordance with the interface line, Figure 4a–c show the decrease of the normalized TERS intensity of WS_2_ in domain A, and Figure 4e–h show the increase of the normalized TERS intensity of MoS_2_ in domain B. Note that, it is different that deduced line with the normalized TERS line profile with the interface line seen by the OM and STM images. Near the spot with tip displacement ≈ 200 nm, the TERS signal shows drastic changes, which means that the actual interface is shifted from the center. Due to the formation of the multi-disorders with crystal alloy, the interface of the lateral heterostructure is not atomically sharp like OM and STM images and introduced the invasion on both sides and exhibited the difference of location with around 250 nm between the interface line of microscopic image and TERS line profile.

Also, Figure 4c and d, which shows a consistent intensity tendency along the tip displacement, imply the possibility of the presence of alloy Mo_1−*x*_W_*x*_S_2_. Since the normalized TERS intensity and WS_2_ PL background suddenly decreased at the position with 200 nm, the signal decreasing region (200 nm ≤ tip displacement < 500 nm) could be considered as defective region or continuous alloy formed region. However, it suggests that the gradual signal diminishing has not been induced with only disorders by means of our high spatial resolution. Moreover, the Raman intensity in Figure 4c depends on the difference between the laser energy and band gap of the semiconductor, which determines the strength of the resonance Raman scattering process, which can also lead to PL intensity change [30], [39], [40]. In other words, there is the possibility with that the formation of continuous alloy and several disorders that cause the noise on signals.

### 3.3 Variations of nanoscale alloy composition at lateral heterostructure

**Figure 5:**
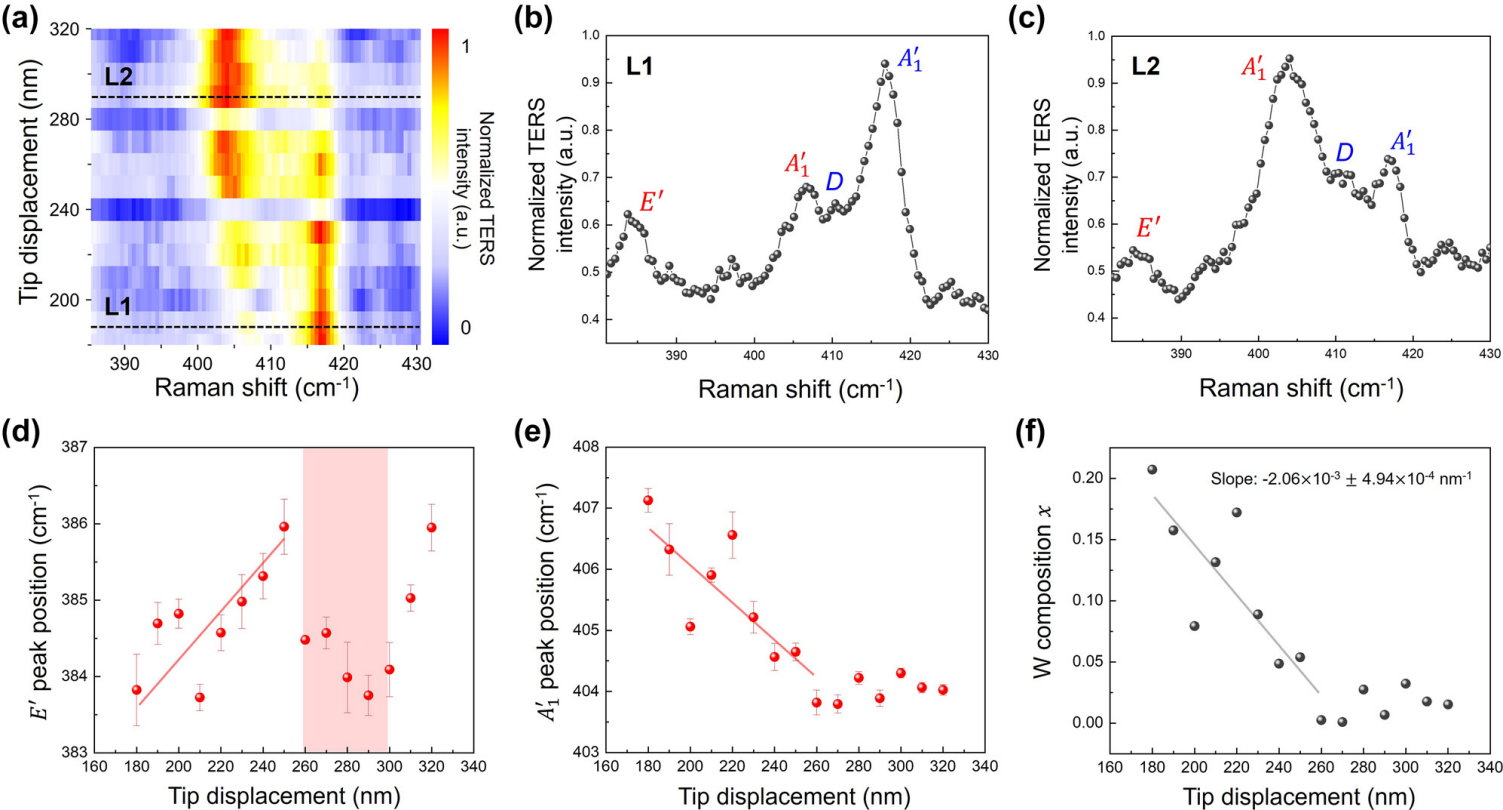
Probing the composition variation of alloy in nanoscale. (a) Normalized TERS spectra of Mo_1−*x*_W_*x*_S_2_ region with respect to the tip displacement from 180 nm to 320 nm. The black dashed lines L1 and L2 indicate the normalized TERS spectra (b) and (c), respectively. Assigned phonon modes with red and blue colors indicate the vibration modes of MoS_2_ and WS_2_, respectively. The graph of variation of (d) *E*′ and (e) $A_{1}^{\prime }$ mode peak position of MoS_2_ along the tip displacement in Mo_1−*x*_W_*x*_S_2_ region. All the peak position and error bar values are plotted from deconvolution results. (f) The graph of change in calculated *W* composition *x* on the basis of (e) in alloy region.

To probe the variations of the alloy characteristics in the nanoscale, we investigated the atomic ratio between Mo and W, the crystal alloy compositions, by the deconvoluted TERS spectra in Figure 3a and b (Figure S3, Supplementary Materials) in the Mo_1−*x*_W_*x*_S_2_ region with drastic change in phonon frequency. Figure 5a clearly shows the intensity changes of the first-order Raman scattering signals along the tip displacement from 170 nm to 320 nm. As shown in Figure 5b and c, which demonstrate the Raman spectra of L1 and L2, the TERS intensity on two different $A_{1}^{\prime }$ modes of MoS_2_ and WS_2_ were reversed. Figure 5d and e shows the deconvolution results along the tip displacement that *E*′ and $A_{1}^{\prime }$ modes frequency of MoS_2_ at the region, which is considered as alloy formed. According to the tip travels from 170 nm to 260 nm position, the frequency of both first-order Raman modes gradually shifted to close to each other due to the composition changes of Mo_1−*x*_W_*x*_S_2_. Over the 260 nm position, most of the peak frequencies maintained the last frequency values of *E*′, and $A_{1}^{\prime }$ , respectively. It implies that the termination of changes in alloy composition, material change from WS_2_ to MoS_2_, and emergence of intact monolayer MoS_2_ ( $\omega_{{A^{\prime }}_{1}}-\omega_{E^{\prime }}<$ 20 cm^−1^). One of the anomalies is that the *E*′ mode frequency in the region highlighted in red in Figure 5d shows a frequency shift at the localized 40 nm area. Since the $A_{1}^{\prime }$ mode frequency did not show any changes after the 260 nm position, which proved the formation of intact MoS_2_, the abrupt *E*′ mode frequency change could be explained by the variations of vacancy density, nanocrystallites domain sizes which directly affect to the phonon frequency of the heterogeneous interface region. Moreover, we set up the function, *y* = *ax* + *b*, for this research that is fit to the model of Chen *et al.* which could be used to calculate the composition value of *W* along the tip displacement in the nanoscale (see details in the Supplementary Materials) [48]. As shown in Figure 5f, the *W* composition *x* converged continuously from 0.2 to 0 within tens of nanometers. In other words, via spectroscopic analysis, we have demonstrated the nanoscale crystal alloy and the gradual variation in *W* composition *x* within the vicinity of the lateral heterostructure interface, within a range of several tens of nanometers. By using TERRS, with high spatial resolution and ultra-high sensitivity on signal, we can explore the nanoscopic nature of the heterogeneous materials, and it can apply in material science and engineering as well as the conventional spectroscopic studies.

## 4 Conclusions

In this research, we have carried out an STM-based TERS experiment to investigate the interfacial nature of the lateral heterostructure of the MoS_2_–WS_2_ monolayer. TERRS line trace analysis across the heterogeneous interface revealed the multi-disorders that are hard to prevent at the TMDs lateral heterostructure. The disorder-induced crystal structure exhibited numerous phonon modes due to the phonon-disorder scattering process based on the phonon confinement effect. Our results provided the spectroscopic evidence of the presence of disorder complexes containing atomic vacancies, crystal edge, and nanocrystallites at the heterojunction interface. In addition, the variation of *L*_*c*_, which could be easily influenced by the disorder concentration and domain sizes, is attributed to the phonon frequency changes along the tip displacement. Furthermore, we obtained the gradual composition change of Mo_1−*x*_W_*x*_S_2_ using spectroscopical information, within the range of tens of nanometers, based on the atomic ratio of the crystal alloy that was spatially resolved. In conclusion, our research on a nondestructive nanoscale imaging system for probing lattice vibrational characteristics at the interface of lateral heterostructure holds promise for advancing the applications and assessment of excitonic functionalities based on bandgap modulation in TMDs lateral heterostructure.

## Supplementary Material

Supplementary Material Details
