# Supplementary material for: Probing the multi-disordered nanoscale alloy at the interface of lateral heterostructure of MoS2–WS2
Source: Nanophotonics. 2024 Jan 19;13(7):1069–77. doi: 10.1515/nanoph-2023-0826 (PMC11501852; doi:10.1515/nanoph-2023-0826)
Supplement: Supplementary file 1 — Supplementary Material Details [file j_nanoph-2023-0826_suppl_001.docx]

**Supplementary Materials**

Probing the Multi-disordered Nanoscale Alloy at the Interface of Lateral Heterostructure of MoS_2_-WS_2_

*Dong Hyeon Kim^a, b, 1^, Chanwoo Lee^b, 1^, Sung Hyuk Kim^a, b, 1^, Byeong Geun Jeong^b^, Seok Joon Yun^c^, Hyeong Chan Suh^a^, Dongki Lee^d^, Ki Kang Kim^b^, and Mun Seok Jeong^a,^**

^a^Department of Physics, Hanyang University, Seoul 04763, Korea

^b^Department of Energy Science, Sungkyunkwan University, Suwon 16419, Korea

^c^Department of Semiconductor, University of Ulsan, Ulsan, 44610, Republic of Korea

^d^Department of Nanotechnology and Advanced Materials Engineering, Sejong University, Seoul 05006, Korea

*Address correspondence to [mjeong@hanyang.ac.kr](mailto:mjeong@hanyang.ac.kr)

KEYWORDS, tip-enhanced Raman spectroscopy (TERS), molybdenum disulfide (MoS_2_), tungsten disulfide (WS_2_), multi-disorder, nanoscale alloy

**Section 1: The identification of TMDs layer number**

**
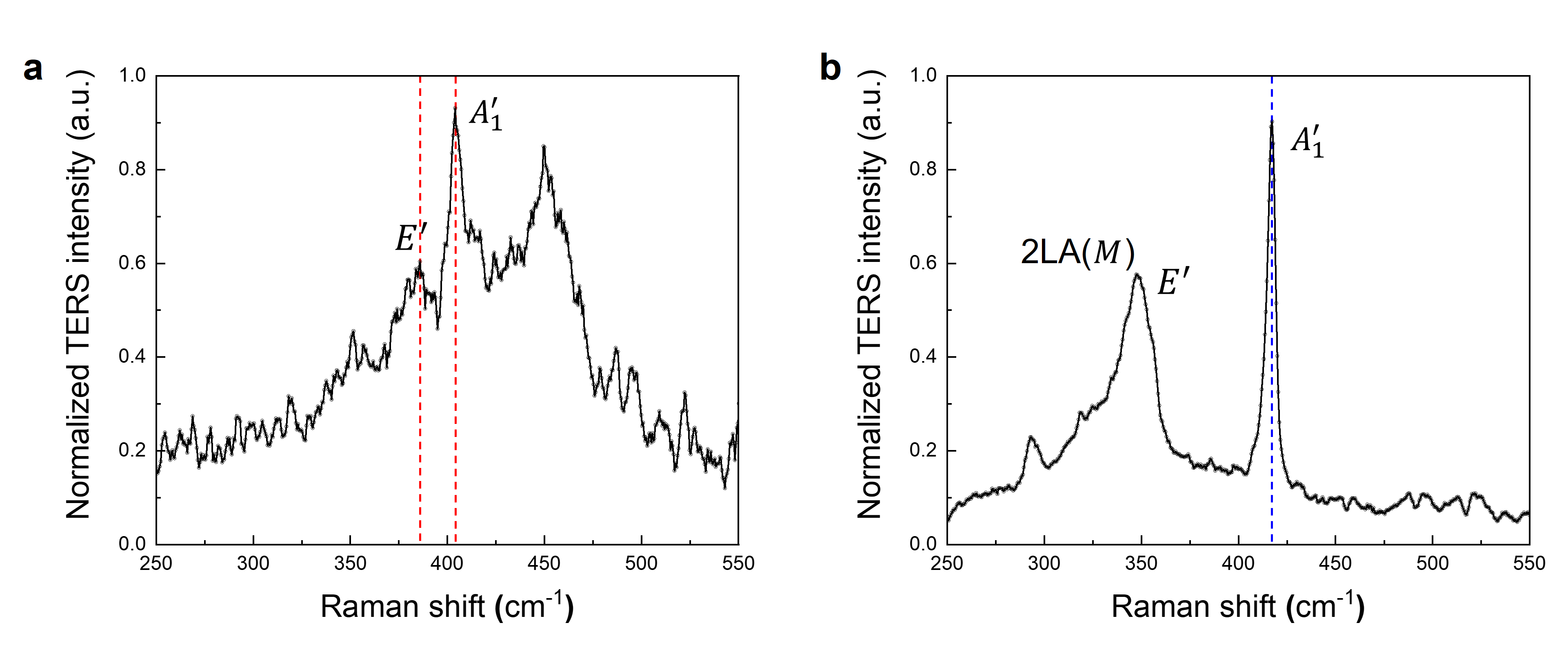
**

Figure S1. Representative TERS spectra of two TMDs. The representative normalized TERS spectrum of (a) MoS_2_ and (b) WS_2_. The prominent Raman modes are marked on each phonon.

Based on the phonon frequency difference of MoS_2_ in Figure S1 (a), ($\omega_{A_{1}^{'}}-\omega_{E^{'}}<$20 cm^-1^), we determined the layer number of MoS_2_ as monolayer. Also, in the normalized TERS spectrum of Figure S1 (b), $A_{1}^{'}$ mode frequency value is about 417 cm^-1^ that implies the monolayer WS_2_.

**Section 2: Strong enhancement effect of TERS**


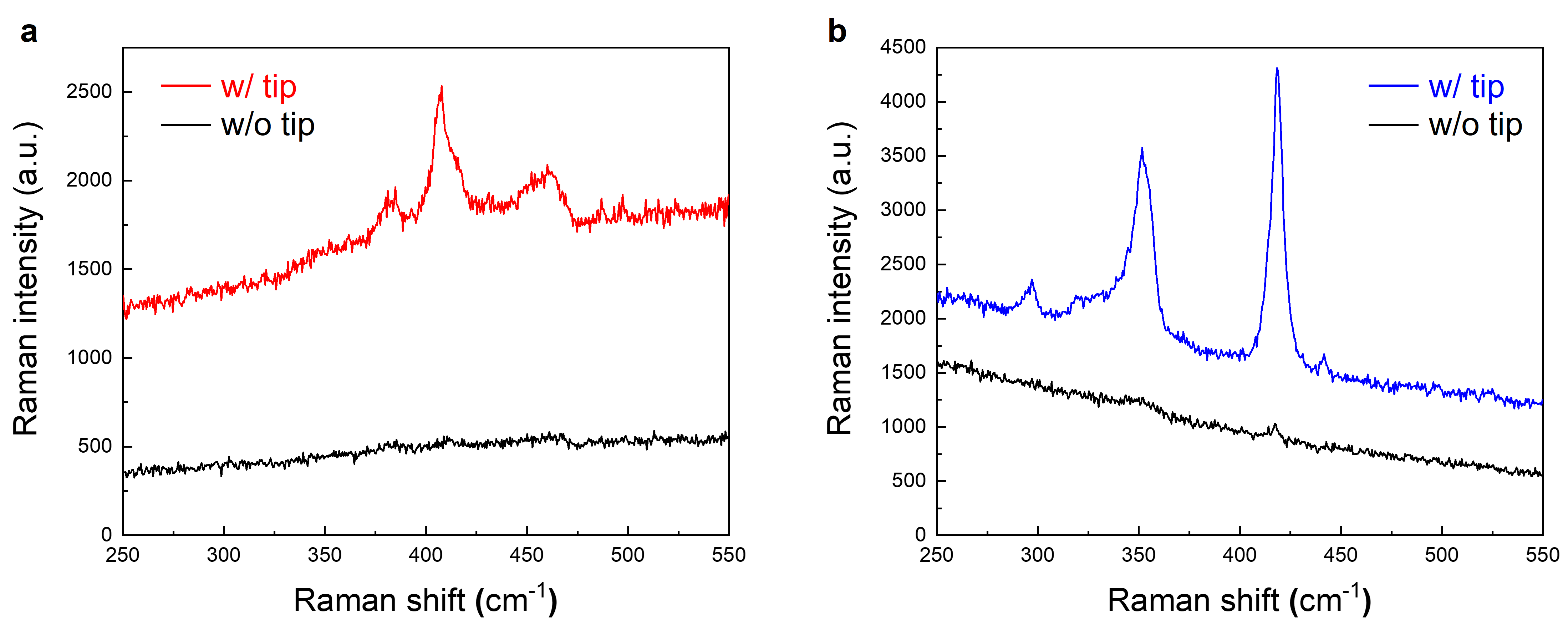


Figure S2. Raman spectra with/without TERS nanotip. Raman spectra of (a) MoS_2_ and (b) WS_2_ with/without TERS nanotip. The black solid line and colored solid line indicate the far-field Raman spectra and TERS spectra, respectively.

The sharp TERS nanotip greatly enhanced the Raman scattering signal in comparison with the far-field Raman scattering signal as shown in Figure S2. The enhancement degree between two materials induced due to the strength difference of resonance Raman scattering process.

**Section 3: TERS nanotip used to Raman measurements**

**
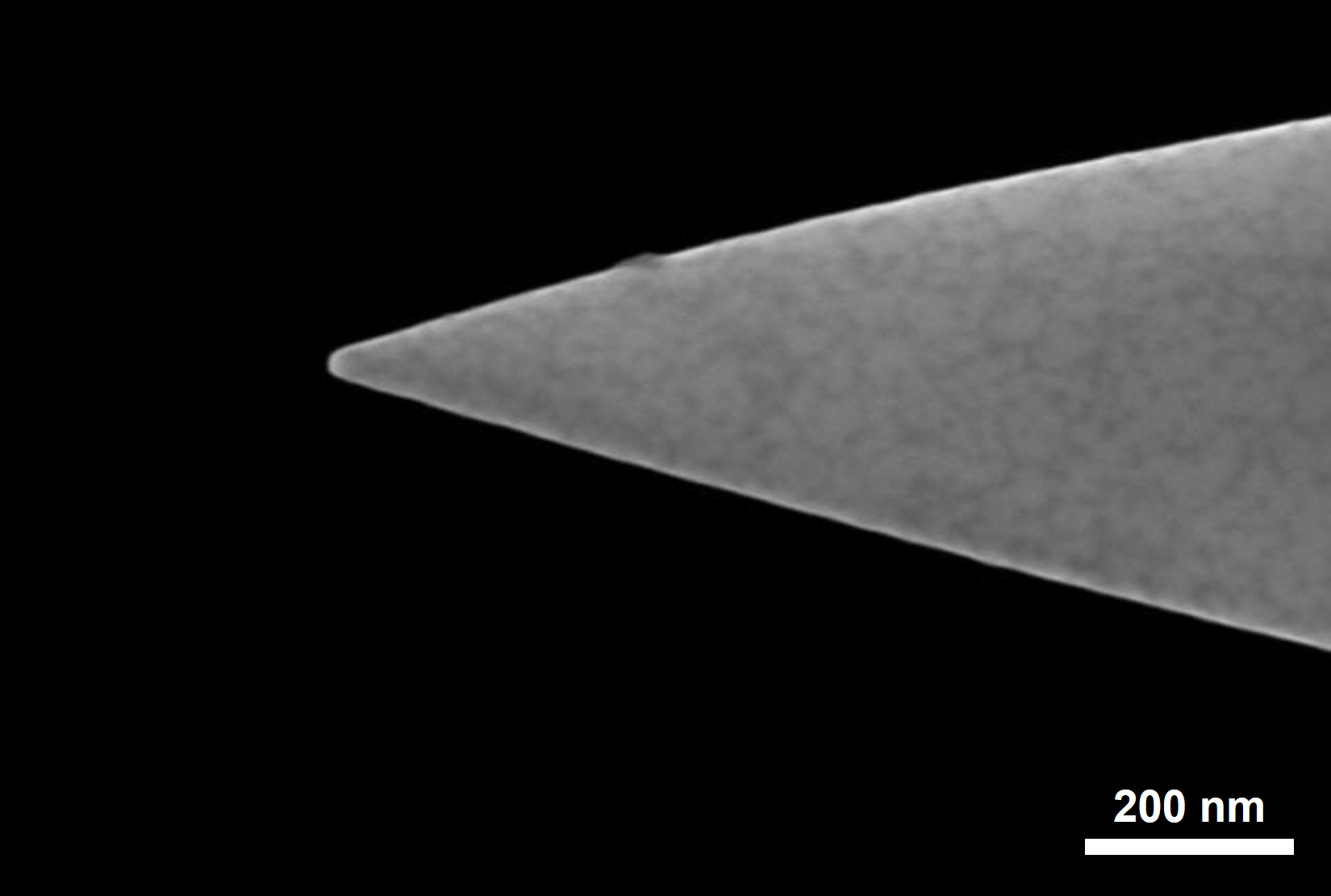
**

Figure S3. Scanning electron microscope (SEM) image of TERS nanotip fabricated by electrochemical etching process. The radius of curvature of nanotip is around 15 nm.

**Section 4: The interface line of lateral heterostructure**


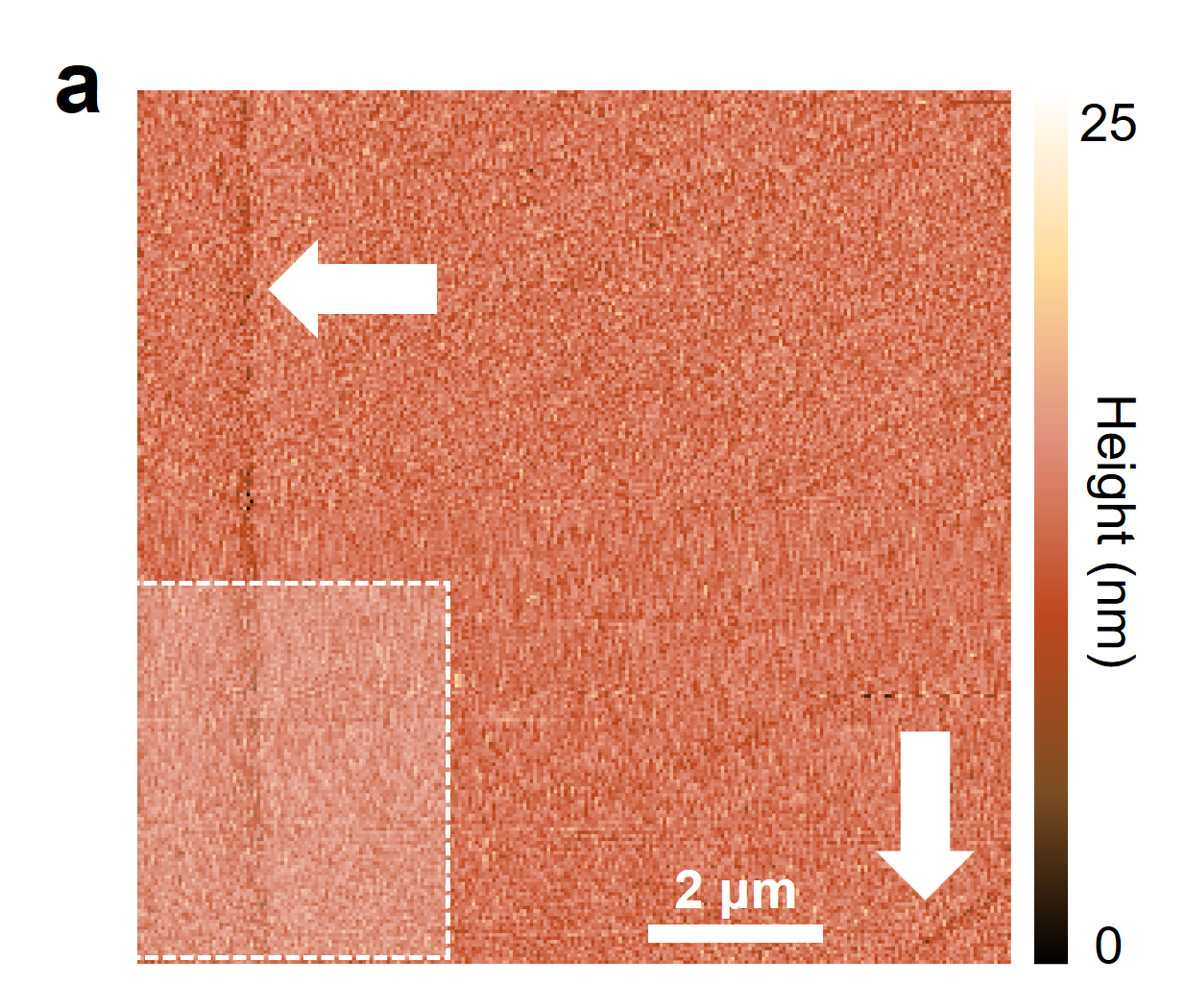


Figure S4. Raw STM image of figure 1d without green dotted lines. The white solid arrows point the interface of the lateral heterostructure.

We marked the interface line of lateral heterostructure by a green dotted line in Figure 1, based on the topographical information. The white solid arrows in Figure S3b indicate the interface line between two TMDs intuitively.

**Section 5: Statistical fitting distribution for deconvolution process**


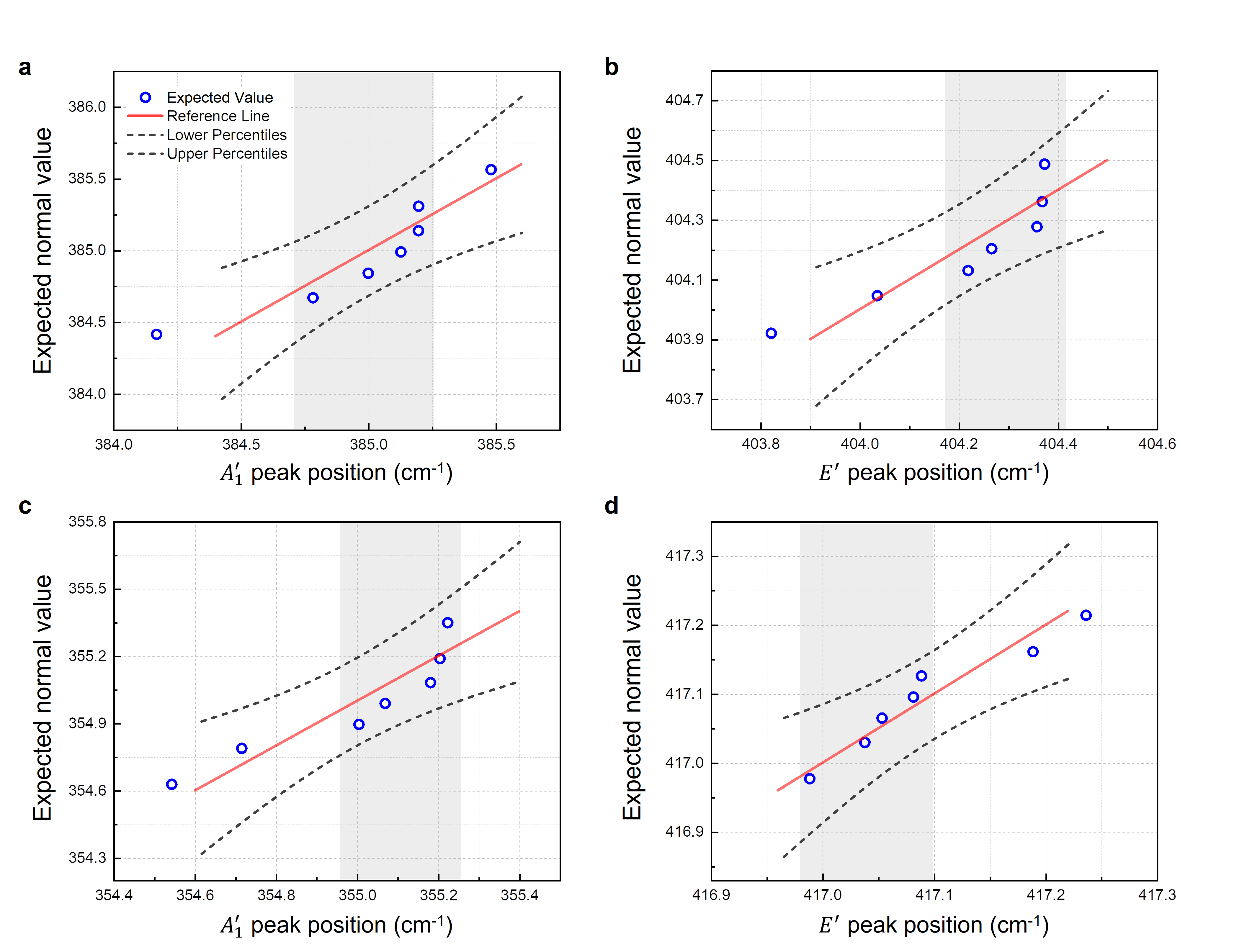


Figure S5. Normal Q-Q plots of the frequency of representative phonon modes. Q-Q plot of deconvoluted phonon mode from random positions in pure (a, b) MoS_2_ and (c, d) WS_2_ regions. Blom score method was used with 95 % confidence level.

To ensure the accuracy of the deconvolution process, we plotted Q-Q plot for normal distribution of deconvolution results. The frequency of representative phonon modes was normally distributed in pure MoS_2_ and WS_2_ regions and increased population could enhance the reliability of plots.

**Section 6: The assignment of phonons of deconvolution results**

Table S1. The assignment of each phonon mode observed in TERS line trace and respective frequency. The peak number is matched with Figure 3a, and the blue color indicates phonon modes of WS_2_, and the red color indicates phonon modes of MoS_2_.


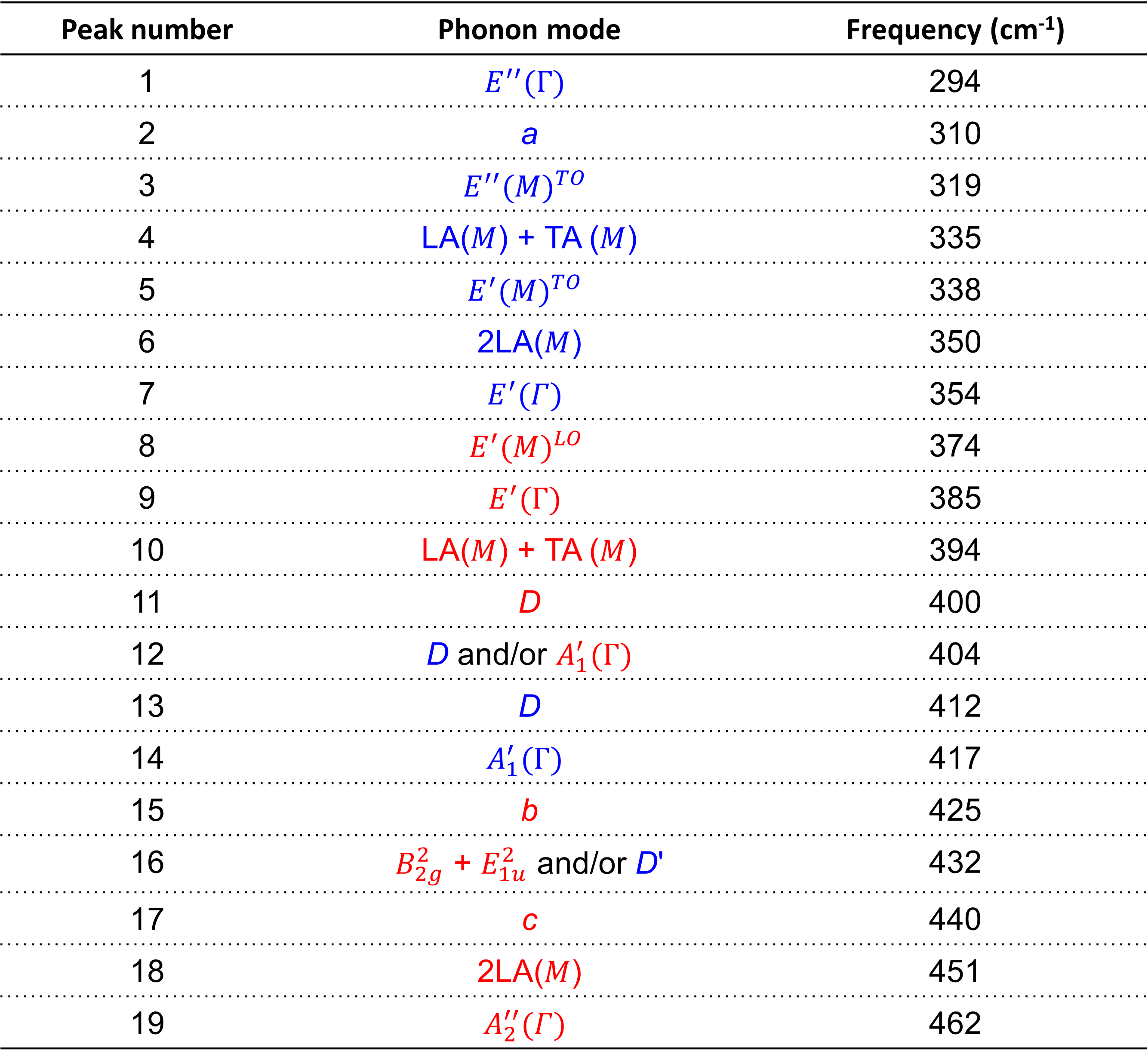


The phonon modes with peak number 2, 15, and 17 will be further investigated.

**Section 7: Calculation of alloy composition with the function based on the model of Chen *et al*.**

We set up the function about the alloy composition, $y=ax+b$, for this research which is based on the study of Chen *et al*. that was the application of modified random-element-isodisplacement (MREI) model. To calculate the W composition $x$ of TMDs alloy, the function was used as follows:

$\omega^{2}=\frac{\left( 1-x \right)\left( 1-\theta_{1}x \right)F_{10}+x\left( 1-\theta_{1}x \right)F_{20}}{m_{s}}$ (1)

$\omega_{A_{1}^{'}}=403.78 {(0.002x^{2}+0.0080x+1)}^{1/2}$ (2)

The coefficients were modified to our research conditions based on the experimental results.
